# Supplementary material for: Adverse childhood experiences, stress impact, and well-being in deaf and hard of hearing adolescents and adolescents with developmental language disorders in special secondary education
Source: PLOS Ment Health. 2025 Dec 5;2(12):e0000466. doi: 10.1371/journal.pmen.0000466 (PMC12798341; doi:10.1371/journal.pmen.0000466)
Supplement: S1 Table — (PDF) [file pmen.0000466.s001.pdf]

Table 1

*ACE Total Tests of Between-Subjects Effects Reference Group - Target Group*

Dependent variable: 16 ACEs total

| Source | Type III Sum of Squares | *df* | Mean square | *F* | Sig. |
|---|---|---|---|---|---|
| Corrected model | 101.539[a] | 2 | 50.770 | 5.458 | .005 |
| Intercept | 772.060 | 1 | 772.060 | 83.003 | <.001 |
| Education practical - theoretical | 14.044 | 1 | 14.044 | 1.510 | .221 |
| RG- TG | 33.638 | 1 | 33.638 | 3.616 | .059 |
| Error | 1953.343 | 210 | 9.302 | | |
| Total | 5423.000 | 213 | | | |
| Corrected total | 2054.883 | 212 | | | |

Note: a. R Squared = .049 (Adjusted *R* Squared = .040). *N* = 213. Adolescents with CP *n* = 127. Reference group, RG *n* = 86. DHH *n* = 32, DLD *n* = 95.
