# Supplementary material for: Adverse childhood experiences, stress impact, and well-being in deaf and hard of hearing adolescents and adolescents with developmental language disorders in special secondary education
Source: PLOS Ment Health. 2025 Dec 5;2(12):e0000466. doi: 10.1371/journal.pmen.0000466 (PMC12798341; doi:10.1371/journal.pmen.0000466)
Supplement: S2 Table — (PDF) [file pmen.0000466.s002.pdf]

Table 2

Stress Impact Tests of Between-Subjects Effects Reference Group - Target Group

Dependent variable: Stress impact					
Source	Type III Sum of Squares	*df*	Mean square	*F*	Sig.
Corrected model	5192.264[a]	2	2596.132	13.076	<.001
Intercept	29852.213	1	29852.213	150.360	<.001
Education practical - theoretical	385.000	1	385.000	1.939	.165
RG- TG	2468.385	1	2468.385	12.433	<.001
Error	37126.689	187	198.538		
Total	170915.000	190			
Corrected total	42318.953	189			

Note: a. R Squared = .123 (Adjusted R Squared = .113). $N = 190$, missing $n = 23$. Adolescents with CP, $n = 114$ (DHH $n = 28$, DLD $n = 86$). Reference group, RG $n = 76$.
