# Supplementary material for: Adverse childhood experiences, stress impact, and well-being in deaf and hard of hearing adolescents and adolescents with developmental language disorders in special secondary education
Source: PLOS Ment Health. 2025 Dec 5;2(12):e0000466. doi: 10.1371/journal.pmen.0000466 (PMC12798341; doi:10.1371/journal.pmen.0000466)
Supplement: S3 Table — (PDF) [file pmen.0000466.s003.pdf]

Table 3

Well-being Tests of Between-Subjects Effects Reference Group - Target Group

Dependent variable: Well-being

Source	Type III Sum of Squares	*df*	Mean square	*F*	Sig.
Corrected model	651.890[a]	2	325.945	3.675	.027
Intercept	179124.035	1	179124.035	2019.707	<.001
Education practical - theoretical	2.003	1	2.003	.023	.881
RG- TG	490.398	1	490.398	5.529	.020
Error	18624.504	210	88.688		
Total	607776.000	213			
Corrected total	19276.394	212			

Note: a. R Squared = .034 (Adjusted R Squared = .025). *N* = 213. Adolescents with CP *n* = 127. Reference group, RG *n* = 86. DHH *n* = 32, DLD *n* = 95.
