# Supplementary material for: Adverse childhood experiences, stress impact, and well-being in deaf and hard of hearing adolescents and adolescents with developmental language disorders in special secondary education
Source: PLOS Ment Health. 2025 Dec 5;2(12):e0000466. doi: 10.1371/journal.pmen.0000466 (PMC12798341; doi:10.1371/journal.pmen.0000466)
Supplement: S4 Table — (PDF) [file pmen.0000466.s004.pdf]

Table 4

ACE Total Tests of Between-Subjects Effects DHH Adolescents - Adolescents with DLD

Dependent variable: 16 ACEs total

Source	Type III Sum of Squares	*df*	Mean square	*F*	Sig.
Corrected model	15.612[a]	2	7.806	.731	.483
Intercept	380.056	1	380.056	35.591	<.001
Education practical - theoretical	3.359	1	3.359	.315	.576
DHH - DLD	14.812	1	14.812	1.387	.241
Error	1324.136	124	10.679		
Total	3916.000	127			
Corrected total	1339.748	126			

Note: a. R Squared = .012 (Adjusted R Squared = -.004). *N* = 127. DHH *n* = 32. DLD *n* = 95.
