# Supplementary material for: Adverse childhood experiences, stress impact, and well-being in deaf and hard of hearing adolescents and adolescents with developmental language disorders in special secondary education
Source: PLOS Ment Health. 2025 Dec 5;2(12):e0000466. doi: 10.1371/journal.pmen.0000466 (PMC12798341; doi:10.1371/journal.pmen.0000466)
Supplement: S5 Table — (PDF) [file pmen.0000466.s005.pdf]

Table 5

Stress Impact Tests of Between-Subjects Effects DHH Adolescents - Adolescents with DLD

Dependent variable: Stress impact					
Source	Type III Sum of Squares	*df*	Mean square	*F*	Sig.
Corrected model	852.744[a]	2	426.372	1.978	.143
Intercept	10000.000	1	10000.000	46.394	<.001
Education practical - theoretical	285.736	1	285.736	1.326	.252
DHH - DLD	364.300	1	364.300	1.690	.196
Error	23925.537	111	215.545		
Total	128220.000	114			
Corrected total	24778.281	113			

Note: a. R Squared = .034 (Adjusted R Squared = .017). N = 114. DHH n = 28. DLD n = 86.
