# Supplementary material for: Adverse childhood experiences, stress impact, and well-being in deaf and hard of hearing adolescents and adolescents with developmental language disorders in special secondary education
Source: PLOS Ment Health. 2025 Dec 5;2(12):e0000466. doi: 10.1371/journal.pmen.0000466 (PMC12798341; doi:10.1371/journal.pmen.0000466)
Supplement: S6 Table — (PDF) [file pmen.0000466.s006.pdf]

Table 6

Well-being Tests of Between-Subjects Effects DHH Adolescents - Adolescents with DLD

Dependent variable: Well-being

Source	Type III Sum of Squares	*df*	Mean square	*F*	Sig.
Corrected model	171.218[a]	2	85.609	.975	.380
Intercept	42443.351	1	42443.351	483.429	<.001
Education practical - theoretical	58.391	1	58.391	.665	.416
DHH - DLD	148.866	1	148.866	1.696	.195
Error	10886.766	124	87.797		
Total	343019.000	127			
Corrected total	11057.984	126			

Note: a. R Squared = .015 (Adjusted R Squared = .000). *N* = 127. DHH *n* = 32. DLD *n* = 95.
