# Supplementary material for: Adverse childhood experiences, stress impact, and well-being in deaf and hard of hearing adolescents and adolescents with developmental language disorders in special secondary education
Source: PLOS Ment Health. 2025 Dec 5;2(12):e0000466. doi: 10.1371/journal.pmen.0000466 (PMC12798341; doi:10.1371/journal.pmen.0000466)
Supplement: S7 Table — (PDF) [file pmen.0000466.s007.pdf]

Table 7

T-Test Comparing Stress Impact

Participants	CP		RG		One-sided *p*	*t*	95% *CI*
	M	SD	M	SD			
Stress impact	30.12	14.81	19.86	13.03	<001**	4.92	[6.1, 14.4]

Participants	DHH		DLD		Two-sided *p*	*t*	95% *CI*
	M	SD	M	SD			
Stress impact	26.21	14.98	31.40	14.61	.108	-1.62	[-11.5, 1.2]

Note: N = 190, missing n = 23. Adolescents with CP, n = 114 (DHH n = 28, DLD n = 86). Reference group, RG n = 76. Equal variances assumed. **p < .001.
