# Supplementary material for: Adverse childhood experiences, stress impact, and well-being in deaf and hard of hearing adolescents and adolescents with developmental language disorders in special secondary education
Source: PLOS Ment Health. 2025 Dec 5;2(12):e0000466. doi: 10.1371/journal.pmen.0000466 (PMC12798341; doi:10.1371/journal.pmen.0000466)
Supplement: S8 Table — (PDF) [file pmen.0000466.s008.pdf]

Table 8

Independent Samples Proportion Test Clinical Scores Stress Impact (PTSD Screener CRIES-13)

Groups	Proportion	Proportion Difference	*z*	One-sided *p*	95% *CI*
CP	.488				
RG	.198	.291	4.31	<.001**	[.16, .40]
				Two-sided *p*	
DHH	.344				
DLD	.537	-.193	-1.89	.059	[-.4, .0]

Note: $N = 190$, missing $n = 23$. Adolescents with CP, $n = 114$ (DHH $n = 28$, DLD $n = 86$). Reference group, RG $n = 76$. Equal variances assumed. $^{**}p < .001$.
