# Supplementary material for: Adverse childhood experiences, stress impact, and well-being in deaf and hard of hearing adolescents and adolescents with developmental language disorders in special secondary education
Source: PLOS Ment Health. 2025 Dec 5;2(12):e0000466. doi: 10.1371/journal.pmen.0000466 (PMC12798341; doi:10.1371/journal.pmen.0000466)
Supplement: S9 Table — (PDF) [file pmen.0000466.s009.pdf]

Table 9

T-Test Comparing Well-being

Participants	CP		RG		One-sided *p*	*t*	95% *CI*
	M	SD	M	SD			
Well-being	51.13	9.37	54.69	9.44	.004*	-2.71	[-6.2, -1.0]
Participants	DHH		DLD		Two-sided *p*	*t*	95% *CI*
	M	SD	M	SD			
Well-being	52.75	7.87	50.58	9.80	.258	1.14	[-1.6, 6.0]

Note: $N = 213$. Adolescents with CP $n = 127$. Reference group, RG $n = 86$. DHH $n = 32$, DLD $n = 95$. Equal variances assumed. $*p < .05$.
