# Supplementary material for: Adverse childhood experiences, stress impact, and well-being in deaf and hard of hearing adolescents and adolescents with developmental language disorders in special secondary education
Source: PLOS Ment Health. 2025 Dec 5;2(12):e0000466. doi: 10.1371/journal.pmen.0000466 (PMC12798341; doi:10.1371/journal.pmen.0000466)
Supplement: S10 Table — (PDF) [file pmen.0000466.s010.pdf]

Table 10

Mediation Effect of Stress Impact on Well-Being

Outcome variable stress impact

Model summary

	R	*R*-sq	MSE	*F*(HC4)	df1	df2	*p*
	.5074	.2575	167.1385	52.5694	1.0000	188.0000	.0000

Model

	Coeff	se(HC4)	*t*	*p*	LLCI	ULCI
constant	14.5498	1.8267	7.9650	.0000	10.9463	18.1532
ACEs	2.5721	.3547	7.2505	.0000	1.8723	3.2719

Outcome variable Well-being

Model summary

	R	*R*-sq	MSE	*F*(HC4)	df1	df2	*p*
	.3063	.0938	82.0666	10.3207	2.0000	187.0000	.0001

Model - Direct effect of ACEs on well-being

	Coeff	se(HC4)	*t*	*p*	LLCI	ULCI
constant	56.9933	1.3733	41.5000	.0000	54.2841	59.7025
ACEs	-.1089	.3076	-.3540	.7238	-.7157	.4979
Stress impact	-.1820	.0483	-3.7663	.0002	-.2773	-.0867

Direct effect of X (ACEs) on Y (well-being)

	Effect	se(HC4)	*t*	*p*	LLCI	ULCI
	-.1089	.3076	-.3540	.7238	-.7157	.4979

Indirect effect(s) of X (ACEs) on Y (well-being)

	Effect	BootSE	BootLLCI	BootULCI
Stress impact	-.4681	.1412	-.7730	-.2169

Bootstrap results for regression model parametrics

Outcome variable stress impact

	Coeff	BootMean	BootSE	BootLLCI	BootULCI
constant	14.5498	14.4915	1.7729	11.1049	17.9967
ACEs	2.5721	2.5845	3344	1.9379	3.2396

Outcome variable well-being

	Coeff	BootMean	BootSE	BootLLCI	BootULCI
constant	56.9933	57.0045	1.3377	54.3718	59.6678
ACEs	-.1089	-.1052	.2911	-.6721	.4599
Stress impact	-.1820	-.1828	.0487	-.2777	-.0888

Note: *N* = 190, missing *n* = 23. Adolescents with CP, *n* = 114 (DHH *n* = 28, DLD *n* = 86). Reference group, RG *n* = 76. Level of confidence for all confidence intervals in output: 95.0000. Number of bootstrap samples for percentile bootstrap confidence intervals: 5000. A heteroscedasticity consistent standard error and covariate matrix estimator was used.
