# Supplementary material for: Adverse childhood experiences, stress impact, and well-being in deaf and hard of hearing adolescents and adolescents with developmental language disorders in special secondary education
Source: PLOS Ment Health. 2025 Dec 5;2(12):e0000466. doi: 10.1371/journal.pmen.0000466 (PMC12798341; doi:10.1371/journal.pmen.0000466)
Supplement: S11 Table — (PDF) [file pmen.0000466.s011.pdf]

Table 11

T-Test Comparing Groups Reporting Zero ACEs on Well-being

Participants	CP		RG		Two-sided *p*	*t*	95% *CI*
	M	SD	M	SD			
Well-being	59.23	8.42	58.90	6.61	.920	.102	[-6.4, 7.1]

Note: N = 23. Adolescents with CP n = 13. Reference group, RG n = 10. Equal variances assumed.
