# Supplementary material for: Adverse childhood experiences, stress impact, and well-being in deaf and hard of hearing adolescents and adolescents with developmental language disorders in special secondary education
Source: PLOS Ment Health. 2025 Dec 5;2(12):e0000466. doi: 10.1371/journal.pmen.0000466 (PMC12798341; doi:10.1371/journal.pmen.0000466)
Supplement: S12 Table — (PDF) [file pmen.0000466.s012.pdf]

Table 13

Moderated Mediation - Moderation Effect of Communication Problems on Mediation ACEs, Stress Impact, and Well-being

Outcome variable stress impact

Model summary

	R	*R*-sq	MSE	*F*(HC4)	df1	df2	*p*
	.5556	.3087	157.2812	26.7038	3.0000	186.0000	.0000

Model

	Coeff	se (HC4)	*t*	*p*	LLCI	ULCI
constant	10.9013	2.4554	4.4398	.0000	6.0573	15.7453
ACEs	2.4745	.6601	3.7490	.0002	1.1724	3.7767
CP-No CP	8.1175	3.6659	2.2143	.0280	8853	15.3496
Int_1	-.2615	.8066	-.3242	-.7462	-1.8528	1.3298

Product terms key Int_1: ACEs x CP-NoCP

Test(s) of highest order unconditional interaction(s)

	R2-chng	*F*(HC4)	df1	df2	*p*
X*W	.0006	.1051	1.0000	186.0000	.7462

Outcome variable Well-being

Model summary

	R	*R*-sq	MSE	*F*(HC4)	df1	df2	*p*
	.3063	.0938	82.0666	10.3207	2.0000	187.0000	.0001

Model

	Coeff	se (HC4)	*t*	*p*	LLCI	ULCI
constant	56.9933	1.3733	41.5000	.0000	54.2841	59.7025
ACEs	-.1089	.3076	-.3540	.7238	-.7157	.4979
Stress impact	-.1820	.0483	-3.7663	.0002	-.2773	-.0867

Direct effect of X (ACEs) on Y (well-being)

	Effect	se(HC4)	*t*	*p*	LLCI	ULCI
	-.1089	.3076	-.3540	.7238	-.7157	.4979

Conditional indirect effects of X (ACEs) on Y (well-being)

Indirect effect of ACEs - stress impact - well-being

CP-No CP	Effect	BootSE	BootLLCI	BootULCI
.0000	-.4503	.1610	-.8140	-.1798
1.0000	-.4027	.1285	-.6841	-.1824

Index of moderated mediation (difference between conditional indirect effects):

	Index	BootSE	BootLLCI	BootULCI
CP-No CP	.0476	.1404	-.2278	.3413

Bootstrap results for regression model parameters

Outcome variable Stress impact

	Coeff	BootMean	BootSE	BootLLCI	BootULCI
Constant	10.9013	10.7840	2.3256	6.2871	15.5671
ACEs	2.4745	2.5092	.5952	1.3217	3.7071

CP-No CP	8.1175	8.1162	3.5231	.9840	14.8691
Int_1	-.2615	-.2711	.7412	-1.7059	1.2397

Outcome variable well-being

	Coeff	BootMean	BootSE	BootLLCI	BootULCI
Constant	56.9933	56.9996	1.3718	54.3366	59.7446
ACEs	-.1089	-.1084	.2920	-.6882	.4626
Stress impact	-.1820	-.1814	.0473	-.2737	-.0880

Note: N = 190, missing n = 23. Adolescents with CP, n = 114 (DHH n = 28, DLD n = 86). Reference group, RG n = 76. Level of confidence for all confidence intervals in output: 95.0000. Number of bootstrap samples for percentile bootstrap confidence intervals: 5000. A heteroscedasticity consistent standard error and covariate matrix estimator was used.
