# Supplementary material for: Adverse childhood experiences, stress impact, and well-being in deaf and hard of hearing adolescents and adolescents with developmental language disorders in special secondary education
Source: PLOS Ment Health. 2025 Dec 5;2(12):e0000466. doi: 10.1371/journal.pmen.0000466 (PMC12798341; doi:10.1371/journal.pmen.0000466)
Supplement: S13 Table — (PDF) [file pmen.0000466.s013.pdf]

Table 14

Comparing Total ACE Prevalence DHH Adolescents - Adolescents with DLD

ACE prevalence	*F*	*t*	*df*	Two-sided *p*	*SE*	95% *CI*
	.579	-1.074	125	.285	.666	[-2.0, .6]

Note: $N = 127$. (DHH $n = 32$, DLD $n = 95$). Equal variances assumed.
