# Supplementary material for: Adverse childhood experiences, stress impact, and well-being in deaf and hard of hearing adolescents and adolescents with developmental language disorders in special secondary education
Source: PLOS Ment Health. 2025 Dec 5;2(12):e0000466. doi: 10.1371/journal.pmen.0000466 (PMC12798341; doi:10.1371/journal.pmen.0000466)
Supplement: S14 Table — (PDF) [file pmen.0000466.s014.pdf]

Table 15

Comparing Total ACE Prevalence Adolescents with CP - Reference Group

ACE prevalence	*F*	*t*	*df*	One-sided *p*	*SE*	95% *CI*
	2.885	3.063	211	.001*	.426	[.5, 2.1]

Note: $N = 213$. Adolescents with CP $n = 127$. Reference group, RG $n = 86$. Equal variances assumed. *$p < .05$.
