# Supplementary material for: Adverse childhood experiences, stress impact, and well-being in deaf and hard of hearing adolescents and adolescents with developmental language disorders in special secondary education
Source: PLOS Ment Health. 2025 Dec 5;2(12):e0000466. doi: 10.1371/journal.pmen.0000466 (PMC12798341; doi:10.1371/journal.pmen.0000466)
Supplement: S15 Table — (PDF) [file pmen.0000466.s015.pdf]

Table 16

Independent Samples Proportion Tests Sexual Abuse

Sexual abuse	CP	RG	Prop. difference	*z*	One-sided *p*	95% *CI*
No	.764	.872	-.108	-1.967	.025*	[-.21, -.00]
1.Have you ever unintentionally seen other people having sex or unintentionally watched porn?	.189	.105	.084	1.669	.048*	[-.02, .18]
2.Did an adult or person older than you ever against your will touch or fondle you or have you touch their body in a sexual way?	.071	.047	.024	.729	.233	[-.05, .09]
3.Did an adult or person older than you ever against your will attempt or actually have intercourse with you?	.031	.000	.031	1.661	.048*	[-.01, .07]
**Sexual abuse**	**DHH**	**DLD**	**Prop. difference**	***z***	**Two-sided *p***	**95% *CI***
No	.781	.758	.023	*.269*	.788	[-.14, .18]
1. Have you ever unintentionally seen other people having sex or unintentionally watched porn?	.188	.189	-.002	*-.025*	.980	[-.15, .17]
2. Did an adult or person older than you ever against your will touch or fondle you or have you touch their body in a sexual way?	.031	.084	-.053	*-1.010*	.313	[-.13, .06]
3. Did an adult or person older than you ever against your will attempt or actually have intercourse with you?	.000	.042	-.042	*-1.179*	.238	[-.09, .05]

Note: $N = 213$. Adolescents with CP $n = 127$. Reference group, RG $n = 86$. DHH $n = 28$, DLD $n = 86$. $^*p < .05$.
