# Supplementary material for: Adverse childhood experiences, stress impact, and well-being in deaf and hard of hearing adolescents and adolescents with developmental language disorders in special secondary education
Source: PLOS Ment Health. 2025 Dec 5;2(12):e0000466. doi: 10.1371/journal.pmen.0000466 (PMC12798341; doi:10.1371/journal.pmen.0000466)
Supplement: S16 Table — (PDF) [file pmen.0000466.s016.pdf]

Table 17

Robust Test of Equality of Means Child Abuse, Household Dysfunction, ACE Total, Stress Impact, Well-being, Reference Group - Target Group

Welch	Statistic[a]	*df*1	*df*2	Sig.
Child abuse	15.669	1	208.091	<.001
Household dysfunction	1.454	1	191.146	.229
16 ACEs total	10.062	1	202.012	.002
Stress impact	25.358	1	174.093	<.001
Well-being	7.341	1	181.719	.007

Note: a. Asymptotically F distributed. Note: $N = 213$. Adolescents with CP $n = 127$. Reference group, RG $n = 86$. DHH $n = 32$, DLD $n = 95$.
