# Supplementary material for: Adverse childhood experiences, stress impact, and well-being in deaf and hard of hearing adolescents and adolescents with developmental language disorders in special secondary education
Source: PLOS Ment Health. 2025 Dec 5;2(12):e0000466. doi: 10.1371/journal.pmen.0000466 (PMC12798341; doi:10.1371/journal.pmen.0000466)
Supplement: S17 Table — (PDF) [file pmen.0000466.s017.pdf]

Table 18

Robust Test of Equality of Means Child Abuse, Household Dysfunction, ACE Total, Stress Impact, Well-being, DHH Adolescents - Adolescents with DLD

Welch	Statistic[a]	*df*1	*df*2	Sig.
Child abuse	1.854	1	59.714	.178
Household dysfunction	1.413	1	68.330	.239
16 ACEs total	1.338	1	61.365	.252
Stress impact	2.557	1	44.954	.117
Well-being	1.601	1	65.945	.210

Note: a. Asymptotically F distributed. $N = 127$. DHH $n = 32$, DLD $n = 95$.
