# Supplementary material for: Adverse childhood experiences, stress impact, and well-being in deaf and hard of hearing adolescents and adolescents with developmental language disorders in special secondary education
Source: PLOS Ment Health. 2025 Dec 5;2(12):e0000466. doi: 10.1371/journal.pmen.0000466 (PMC12798341; doi:10.1371/journal.pmen.0000466)
Supplement: S18 Table — (PDF) [file pmen.0000466.s018.pdf]

Table 19

Independent Samples Effect Sizes Child Abuse, Household Dysfunction, ACE Total, Stress Impact, Well-being, Reference Group - Target Group

			95% Confidence Interval	
Hedges'*d*	Standardizer[a]	Point estimate	Lower	Upper
Child abuse	1.332	.523	.245	.800
Household dysfunction	1.303	.165	-.108	.438
16 ACEs total	3.064	.426	.150	.702
Stress impact	14.182	.724	.425	1.021
Well-being	9.429	-.378	-.652	-.102

Note: a. The denominator used in estimating the effect sizes. Note: $N = 213$. Adolescents with CP $n = 127$. Reference group, RG $n = 86$. DHH $n = 32$, DLD $n = 95$.
