# Supplementary material for: Adverse childhood experiences, stress impact, and well-being in deaf and hard of hearing adolescents and adolescents with developmental language disorders in special secondary education
Source: PLOS Ment Health. 2025 Dec 5;2(12):e0000466. doi: 10.1371/journal.pmen.0000466 (PMC12798341; doi:10.1371/journal.pmen.0000466)
Supplement: S19 Table — (PDF) [file pmen.0000466.s019.pdf]

Table 20

Independent Samples Effect Sizes Child Abuse, Household Dysfunction, ACE Total, Stress Impact, Well-being, DHH Adolescents - Adolescents with DLD

Hedges'*d*	Standardizer[a]	Point estimate	95% Confidence Interval	
			Lower	Upper
Child abuse	1.462	-.260	-.659	.140
Household dysfunction	1.342	-.213	-.612	.186
16 ACEs total	3.279	-.218	-.617	.181
Stress impact	14.802	-.350	-.775	.077
Well-being	9.414	.231	-.169	.629

Note: a. The denominator used in estimating the effect sizes. $N = 127$. DHH $n = 32$, DLD $n = 95$.
