# Supplementary figures and images for: Adverse childhood experiences, stress impact, and well-being in deaf and hard of hearing adolescents and adolescents with developmental language disorders in special secondary education
Source: PLOS Ment Health. 2025 Dec 5;2(12):e0000466. doi: 10.1371/journal.pmen.0000466 (PMC12798341; doi:10.1371/journal.pmen.0000466)

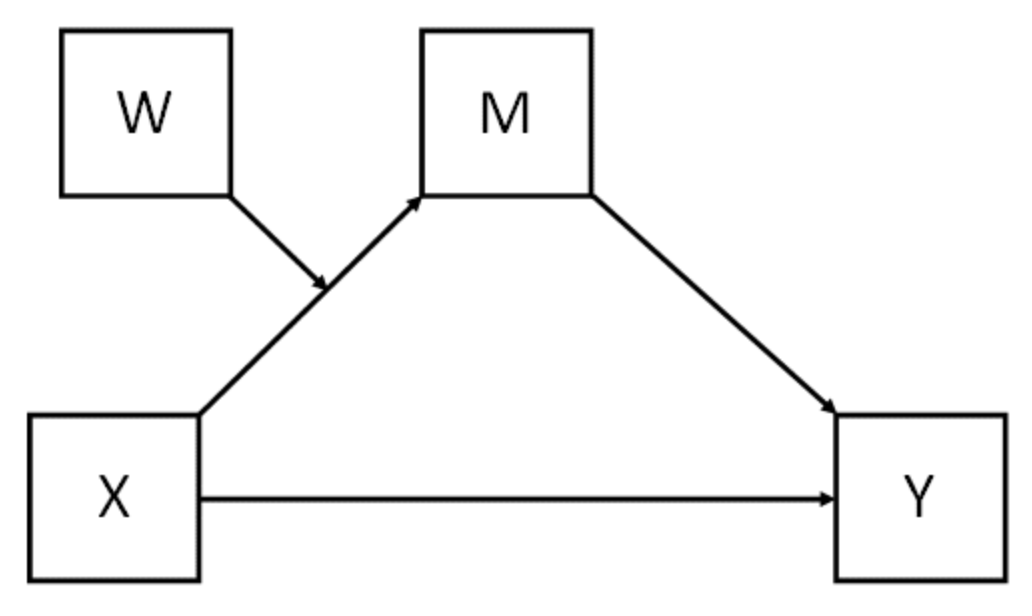

Supplement: S1 Fig — (TIF) [file pmen.0000466.s020.tif]
