# Supplementary material for: Adverse childhood experiences, stress impact, and well-being in deaf and hard of hearing adolescents and adolescents with developmental language disorders in special secondary education
Source: PLOS Ment Health. 2025 Dec 5;2(12):e0000466. doi: 10.1371/journal.pmen.0000466 (PMC12798341; doi:10.1371/journal.pmen.0000466)
Supplement: Attachment — Submitted filename: Response to reviewers.docx [file pmen.0000466.s022.docx]

L. Martijn, MSc.

VierTaal College Amsterdam, Radboud University Nijmegen

Len.martijn@ru.nl

Amsterdam, October 3, 2025

Lambert Zixin Li, Ph.D.
Academic Editor
PLOS Mental Health

Subject: Response to reviewers and revised manuscript - Adverse childhood experiences, stress impact, and well-being in deaf and hard of hearing adolescents and adolescents with developmental language disorders in special secondary education (PMEN-D-25-00299).

Dear Dr. Zixin,

We sincerely thank you and the reviewers for your time and effort in reviewing our manuscript, Adverse childhood experiences, stress impact, and well-being in deaf and hard of hearing adolescents and adolescents with developmental language disorders in special secondary education (PMEN-D-25-00299). The feedback has been very helpful, and we have worked hard to address each of the comments to improve the quality of our paper.

*Response concerning journal requirements:*

1. Participant Consent

In the method section, we added the following lines:

'To participate, written, informed, and active consent was obtained from all students. If a student was under 16 years of age, written, informed, and active consent was also obtained from their parents or guardians.'

2. Competing interests

A competing interests statement is provided as a separate file, in which the authors declare that they have no competing interests.

3. Financial disclosure statement

A full-sentence financial disclosure statement is included as a separate file.

'The authors confirm that this research received no external financial support. All research-related costs were covered by Stichting VierTaal in Amsterdam, the Netherlands.

Authors' salaries were provided by their employing institutions: H.E.T. Knoors, C.T.W.M. Vissers, and D. Hermans from Koninklijke Kentalis in Utrecht, the Netherlands; L. Martijn from Stichting VierTaal in Amsterdam, the Netherlands.

The institutions that employed the researchers had no role in the study design, data collection and analysis, decision to publish, or manuscript preparation.'

4. Data repository

A repository named: 'Adverse childhood experiences, stress impact, and well-being in deaf and hard of hearing adolescents and adolescents with developmental language disorders in special secondary education' at <https://doi.org/10.34973/dgjh-ja34> is assigned by Radboud University Nijmegen to this study and will be activated within the next two weeks. Supplementary materials, original anonymized data files, and outputs will be available for open access in this repository.

5. Supporting information

The following three supporting information files will be uploaded:

- Supporting information_Supplementary material_WithTrackChanges.doc
- Supporting information_Supplementary material.docx

The figures have been converted using the required Preflight Analysis and Conversion Engine (PACE) digital diagnostic tool and will be uploaded.

- Supporting information_Fig1.tiff
- Supporting information_Fig2.tiff

*Response to reviewer #1*

*1. The paper lacks a clear and scientifically sound methodology which can substantiate the quality of the study.*

Thank you for bringing your concern about the methodology to our attention. We agree that vital information was missing from the method section. We have now improved the method section by adding information we hope you were referring to.

- Research design: We now indicate that this study concerns a cross-sectional design (p. 8): 'For this cross-sectional study, a correlational research design was used to compare DHH adolescents with adolescents with DLD.'
- Sample selection and size: An a priori power analysis is included (p. 8): 'An a priori power analysis was conducted using G*Power 3.1, which indicated that per group, 28 participants were needed to detect a large effect size (Cohen's d = .8), with 90% power using a one-sided independent samples t-test with alpha at .05.'
- Data collection procedure: The description of the participant and the parent/guardian type of consent was supplemented with missing information (p. 9): 'To participate, written, informed, and active consent was obtained from all students. If a student was under 16 years of age, written, informed, and active consent was also obtained from their parents or guardians.'
- Variables and measurement: Additional information about the ACE categories is included (p.11): 'To evaluate the cumulative exposure to ACEs, this study calculates the Total ACE score for adolescents by summing the items from the categories Child Abuse, Household Dysfunction, and the six additional ACEs. This created an ACE screener with 16 items in total, with answer options of 'yes' or 'no' (e.g., 'Were your parents ever separated or divorced?'), and scores ranging from 0 to 16.'
- Data analysis: The handling of missing data and outliers is described (pp. 12-13): 'Outliers were kept, preserving the dataset's integrity and ensuring it accurately represents the data.'

We sincerely hope this additional information addresses your feedback.

*2. The paper lacks a theoretical framework for the study.*

We aim to address this feedback as thoroughly as possible, as we have conscientiously made additions to the introduction and the current study sections to establish the theoretical framework better:

Page 3 ' To contextualize this focus, it is essential to understand what ACEs entail and why they matter. ACEs encompass a range of potentially traumatic events occurring during childhood and adolescence, such as maltreatment, household dysfunction, and economic hardship. ACE research has demonstrated that childhood adversities significantly contribute to negative physical and mental health outcomes throughout life [1-3]. While exposure to a single adverse event can elicit significant stress, there is a well-established theoretical framework suggesting that the cumulative burden of multiple ACEs is most strongly associated with long-term detrimental effects on health and well-being [19, 3]. This association between ACE accumulation and adverse health and well-being outcomes has been well documented in research [1, 20]. Some researchers contend that, in addition to the theoretical framework, this association between ACEs and negative health and well-being outcomes is mediated by the impact on one’s stress system. Accumulating ACEs may dysregulate an individual’s stress regulation and response system. Prolonged and excessive activation of the stress regulation and response system can lead to maladaptive responses to subsequent stressors and become ingrained in the child's neurobiological development [21, 22]. When ingrained, it can elevate the risk of physical and mental health problems, and impair cognitive and social functioning, all of which may negatively affect well-being [22-24]. While considerable research exists on ACEs, stress impact, and overall well-being, the interplay among these factors is rarely examined [25]'.

Page 7 ' Finally, this research focuses on the relationship between the accumulation of ACEs, stress impact, and the well-being of adolescents. This is examined through a theoretical framework that posits stress as a mediator between adversity and well-being. Can the accumulation of ACEs be directly associated with reduced well-being in adolescents, or is it the stress evoked by ACEs that potentially affects adolescents' well-being?'

*3. There is no proper description about the researchers qualification, sampling and data collection process.*

Thank you for pointing out this missing information. All test administration was carried out by the first author, and their qualifications are now included on page 9 and 10:

' All 16 special secondary schools for students with CP in the Netherlands were approached to have their students participate in this study. Ten of these schools agreed to participate. In addition, seven mainstream secondary schools took part in this study. Participant recruitment occurred in classrooms from September 2, 2021, to September 28, 2022, through online video or in-person meetings, during which the first author explained the research project. Adolescents were offered 10 euros for their participation. The class mentor informed all parents via email or a paper letter with a link to an infographic and a video explaining the research project in Sign Supported Dutch and Sign Language of the Netherlands. To participate, written, informed, and active consent was obtained from all students. If a student was under 16 years of age, written, informed, and active consent was also obtained from their parents or guardians. The only exclusion criterion was a nonverbal IQ score of less than 80. The first author, a licensed and registered behavioral specialist, administered the data collection. Data collection took place between 2021 and 2023 and involved four student questionnaires. All questionnaires were read aloud in spoken Dutch and administered individually by the first author in a private room at each participating student's school. If DHH students received education supported with Sign Language of the Netherlands or Sign Supported Dutch, or when parents or students preferred, the test administration was supported by a hired, qualified, independent sign language interpreter. Before administering the questionnaires, students were informed of confidentiality rules. All information provided by the students was kept confidential, except in cases where there were signs of physical, emotional, or mental harm or distress. In these cases, professional standards required the first author to subsequently involve the school counselor and transfer responsibility for the student, following the protocol for domestic violence or child abuse. Within a month after participating, students and parents of students under 16 years received a report containing the results of the participant questionnaires, advice based on those results, contact information for professional healthcare services, and the first author's contact information for questions.'

*4. Discussion section lacks support/ refute of other research adequately.*

Thank you for bringing this to our attention. We tried to address this concern within the discussion section on page 22: 'Consistent with the present study, a systematic scoping review conducted by Lam et al^25^ sought to expand the framework of adverse childhood experiences (ACEs) and their psychosocial outcomes by identifying potential links and mechanisms that might attenuate this association. Their review highlighted several studies that incorporated moderators, such as a child’s race or sex assigned at birth, as well as mediators, including self-esteem and sleep problems. The broad range of identified moderating and mediating factors within the studies underscores the complexity of establishing a comprehensive framework. Although this body of work demonstrates considerable variability in the mediators explored, the findings of the current study emphasize that the stress elicited by ACEs may constitute a crucial mediating factor in the pathway linking ACEs to psychosocial outcomes.'

*5. This paper needs to include more recent citations.*

Thank you for this suggestion. We have added the following additional references relevant to this study:

- Arts E, Orobio de Castro B, Luteijn E, Elsendoorn B, Vissers CTWM. Improving social emotional functioning in adolescents with Developmental Language Disorders: A mini review and recommendations. Frontiers in psychiatry [Internet]. 2022;13:966008. Available from: <http://dx.doi.org/10.3389/fpsyt.2022.966008>
- de Jong TJ, van der Schroeff MP, Stapersma L, Vroegop JL. A systematic review on the impact of auditory functioning and language proficiency on psychosocial difficulties in children and adolescents with hearing loss. International Journal of Audiology [Internet]. 2024;63(9):675–85. Available from: <https://dx.doi.org/10.1080/14992027.2023.2261074>
- Khalid U, Majeed N, Chovaz CJ, Choudhary FR, Munawa K. Psychological well-being and mental health risks in deaf and hard of hearing youth: a systematic review. European Child & Adolescent Psychiatry [Internet]. 2025;1–15. Available from: <http://dx.doi.org/10.1007/s00787-025-02795-6>
- Lam N, Fairweather S, Lewer D, Prescott M, Undugoda P, Dickerson J, et al. The association between adverse childhood experiences and mental health, behaviour, and educational performance in adolescence: A systematic scoping review. PLOS Mental Health [Internet]. 2024;1(5):e0000165. Available from: <http://dx.doi.org/10.1371/journal.pmen.0000165>
- Makhoul Khoury S, Cohen A, Fabris MA, Gur A. Neglect of Children with Disabilities: A Scoping Review. Children (Basel, Switzerland) [Internet]. 2025;12(3). Available from: [<https://doi.org/10.3390/children12030386>](http://dx.doi.org/10.3390/children12030386)
- Nightingale R, Hollyer A, Mahon M. Deaf children’s experiences of hearing in everyday life: a systematic review. Journal of deaf studies and deaf education [Internet]. 2025; Available from: <http://dx.doi.org/10.1093/jdsade/enaf035>

*Response to reviewer #2*

*1: For framing, a brief addition to the Introduction would suffice: 1. add 2–3 sentences summarizing developmental considerations for DHH and DLD in adolescence (e.g., rising social/academic demands, later identification in DLD, visibility and accommodation issues in DHH), and state whether you conceptualize their impact on CP as developmentally stable across ages 12–17 or likely to intensify with age.*

We have addressed this question by adding the following text in the current study section (p. 6). We hope this meets your expectations: 'During adolescence, individuals face increasing academic demands along with greater parental expectations for independence. At the same time, social demands intensify as peer interactions grow more complex. These rising expectations across academic, family, and social settings may cause increasing feelings of stress because challenges in language comprehension, communication modalities, and auditory processing may limit participation in class, interactions with parents and peers, and the ability to advocate for oneself [30-33]'.

*2. ACE Composition — Justify aggregating child abuse, household dysfunction, and added community/socioeconomic items into one ACE index. Report category-level results and run sensitivity analyses (abuse-only, household-dysfunction-only, ACE total excluding household dysfunction) to test mediation robustness.*

We sincerely apologize for not providing a sufficient explanation about the aggregated categories of the ACE questionnaire used. We have included additional information in the instrument section regarding Adverse Childhood Experiences page 11: ' These six ACEs are unrelated and, hence, do not form a category. To evaluate the cumulative exposure to ACEs, this study calculates the Total ACE score for adolescents by summing the items from the categories Child Abuse, Household Dysfunction, and the six additional ACEs'. The analyses concerning the ACE categories were included.

**Table 2. Means, Standard Deviations, and T-Tests of ACE Categories and Total ACE Prevalence**

| Participants | RG | | | CP | | One-sided *p* | *t* | 95% CI |
| --- | --- | --- | --- | --- | --- | --- | --- | --- |
| ACEs | M | | SD | M | SD |  |  |  |
| Child abuse | .744 | | 1.108 | 1.441 | 1.457 | <.001** | 3.759 | [.3, 1.1] |
| Household dysfunction | 1.186 | | 1.242 | 1.402 | 1.335 | .118 | 1.189 | [-.1, .6] |
| Total (16) ACEs | 3.198 | | 2.717 | 4.504 | 3.261 | .001* | 3.063 | [.5, 2.1] |
| Participants | | DHH | | DLD | | Two-sided *p* | *t* | 95% CI |
| ACEs | | M | SD | M | SD |  |  |  |
| Child abuse | | 1.156 | 1.322 | 1.537 | 1.493 | .202 | -1.282 | [-1.0, .2] |
| Household dysfunction | | 1.188 | 1.091 | 1.474 | 1.405 | .296 | -1.049 | [-.8, .3] |
| Total (16) ACEs | | 3.969 | 2.901 | 4.684 | 3.368 | .285 | -1.074 | [-2.0, .5] |

Note: *N* = 213. CP *n* = 127. RG *n* = 86. DHH *n* = 32. DLD *n* = 95**p* < .05. ***p* < .001.

The stress impact is measured with the CRIES-13 (a PTSD screener). 'The CRIES-13 was administered after the ACE questionnaire was completed. As the questions refer to stressful events, the CRIES-13 was not administered to adolescents who reported zero ACEs. Adolescents who reported ACEs were instructed to answer the CRIES-13 questions about the ACEs, to which they responded 'yes' (p. 11). This resulted in some adolescents filling out the CRIES-13 about 10 ACEs of 2 different ACE categories, while others may have filled out the CRIES-13 about one single ACE. Unfortunately, this makes it impossible to analyze the stress impact of the two ACE categories or conduct sensitivity and mediation analyses with the categories. We share the reviewer's interest in this information, which could have been a valuable addition to this paper.

*3. Covariate Control — Since the three groups differ demographically, include key covariates in main comparisons and in mediation/moderation models (e.g., age, sex, educational track, living with family; consider school clustering).*

We appreciate your concern about the robustness of our statistical analysis. The demographic table (Table 1, pp. 8-9) has been updated. The 'Family Living Situation' has been removed because one of these family living situations, namely, having divorced parents, is also one of the ACEs in the ACE questionnaire.

**Table 1. Demographic Characteristics of Participants**

| Participants | CP | RG |  |  | DHH | DLD |  |  |
| --- | --- | --- | --- | --- | --- | --- | --- | --- |
| Demographics | % | % | *z* | Two-sided *p* | % | % | *z* | Two-sided *p* |
| **Gender** |  |  |  |  |  |  |  |  |
| Male | 62.99 | 62.79 | .030 | .976 | 65.63 | 62.11 | .357 | .721 |
| Female | 28.35 | 33.72 | -.836 | .403 | 25.00 | 29.47 | -.486 | .627 |
| Neutral/no ans. | 8.66 | 3.49 | 1.495 | .135 | 9.38 | 8.42 | .166 | .868 |
| **Education** |  |  |  |  |  |  |  |  |
| Vocational | 33.86 | 23.26 | -1.664 | .096 | 31.25 | 34.74 | .360 | .718 |
| Vocational (basic-advanced) | 52.76 | 10.47 | 6.321 | <.001** | 40.63 | 56.84 | -1.589 | .112 |
| General vocational | 9.45 | 25.58 | -3.154 | .002* | 18.75 | 6.32 | 2.080 | .038* |
| Senior general | 3.94 | 40.70 | 6.740 | <.001** | 9.38 | 2.11 | -1.829 | .067 |

Note: *N* = 213. CP *n* = 127. RG *n* = 86. DHH *n* = 32. DLD *n* = 95. **p* < .05. ***p* < .001.

For the demographic variables that differ significantly, the levels of education, ANCOVAs are applied. We took your advice and grouped educational attainment into two categories: practical attainment and theoretical attainment. ANCOVAs are conducted for the main group comparisons, comparing the reference group with adolescents with CP and DHH adolescents with adolescents with DLD, regarding ACE prevalence, stress impact, and well-being (see supplemental material, Tables 8-13). After controlling for covariates, there are no statistically significant differences between groups in ACEs, stress impact, or well-being.

Table 8

*Tests of Between-Subjects Effects Reference Group - Target Group*

| Dependent variable: 16 ACEs total | | | | | |
| --- | --- | --- | --- | --- | --- |
| Source | Type III Sum of Squares | df | Mean square | *F* | Sig. |
| Corrected model | 101.539^a^ | 2 | 50.770 | 5.458 | .005 |
| Intercept | 772.060 | 1 | 772.060 | 83.003 | <.001 |
| Education  practical - theoretical | 14.044 | 1 | 14.044 | 1.510 | .221 |
| RG- TG | 33.638 | 1 | 33.638 | 3.616 | .059 |
| Error | 1953.343 | 210 | 9.302 |  |  |
| Total | 5423.000 | 213 |  |  |  |
| Corrected total | 2054.883 | 212 |  |  |  |

Note: a. R Squared = .049 (Adjusted *R* Squared = .040). *N* = 213. Adolescents with CP *n* = 127. Reference group, RG *n* = 86. DHH *n* = 32, DLD *n* = 95.

Table 9

*Tests of Between-Subjects Effects Reference Group - Target Group*

| Dependent variable: Stress impact | | | | | |
| --- | --- | --- | --- | --- | --- |
| Source | Type III Sum of Squares | df | Mean square | *F* | Sig. |
| Corrected model | 5192.264^a^ | 2 | 2596.132 | 13.076 | <.001 |
| Intercept | 29852.213 | 1 | 29852.213 | 150.360 | <.001 |
| Education  practical - theoretical | 385.000 | 1 | 385.000 | 1.939 | .165 |
| RG- TG | 2468.385 | 1 | 2468.385 | 12.433 | <.001 |
| Error | 37126.689 | 187 | 198.538 |  |  |
| Total | 170915.000 | 190 |  |  |  |
| Corrected total | 42318.953 | 189 |  |  |  |

Note: a. R Squared = .123 (Adjusted R Squared = .113). *N* = 213. Adolescents with CP *n* = 127. Reference group, RG *n* = 86. DHH *n* = 32, DLD *n* = 95.

Table 10

*Tests of Between-Subjects Effects Reference Group - Target Group*

| Dependent variable: Well-being | | | | | |
| --- | --- | --- | --- | --- | --- |
| Source | Type III Sum of Squares | df | Mean square | *F* | Sig. |
| Corrected model | 651.890^a^ | 2 | 325.945 | 3.675 | .027 |
| Intercept | 179124.035 | 1 | 179124.035 | 2019.707 | <.001 |
| Education  practical - theoretical | 2.003 | 1 | 2.003 | .023 | .881 |
| RG- TG | 490.398 | 1 | 490.398 | 5.529 | .020 |
| Error | 18624.504 | 210 | 88.688 |  |  |
| Total | 607776.000 | 213 |  |  |  |
| Corrected total | 19276.394 | 212 |  |  |  |

Note: a. R Squared = .034 (Adjusted R Squared = .025). *N* = 213. Adolescents with CP *n* = 127. Reference group, RG *n* = 86. DHH *n* = 32, DLD *n* = 95.

Table 11

*Tests of Between-Subjects Effects DHH Adolescents - Adolescents with DLD*

| Dependent variable: 16 ACEs total | | | | | |
| --- | --- | --- | --- | --- | --- |
| Source | Type III Sum of Squares | df | Mean square | *F* | Sig. |
| Corrected model | 15.612^a^ | 2 | 7.806 | .731 | .483 |
| Intercept | 380.056 | 1 | 380.056 | 35.591 | <.001 |
| Education  practical - theoretical | 3.359 | 1 | 3.359 | .315 | .576 |
| DHH - DLD | 14.812 | 1 | 14.812 | 1.387 | .241 |
| Error | 1324.136 | 124 | 10.679 |  |  |
| Total | 3916.000 | 127 |  |  |  |
| Corrected total | 1339.748 | 126 |  |  |  |

Note: a. R Squared = .012 (Adjusted R Squared = -.004). *N* = 127. DHH *n* = 32. DLD *n* = 95.

Table 12

*Tests of Between-Subjects Effects DHH Adolescents - Adolescents with DLD*

| Dependent variable: Stress impact | | | | | |
| --- | --- | --- | --- | --- | --- |
| Source | Type III Sum of Squares | df | Mean square | *F* | Sig. |
| Corrected model | 852.744^a^ | 2 | 426.372 | 1.978 | .143 |
| Intercept | 10000.000 | 1 | 10000.000 | 46.394 | <.001 |
| Education  practical - theoretical | 285.736 | 1 | 285.736 | 1.326 | .252 |
| DHH - DLD | 364.300 | 1 | 364.300 | 1.690 | .196 |
| Error | 23925.537 | 111 | 215.545 |  |  |
| Total | 128220.000 | 114 |  |  |  |
| Corrected total | 24778.281 | 113 |  |  |  |

Note: a. R Squared = .034 (Adjusted R Squared = .017). *N* = 127. DHH *n* = 32. DLD *n* = 95.

Table 13

*Tests of Between-Subjects Effects DHH Adolescents - Adolescents with DLD*

| Dependent variable: Well-being | | | | | |
| --- | --- | --- | --- | --- | --- |
| Source | Type III Sum of Squares | df | Mean square | *F* | Sig. |
| Corrected model | 171.218^a^ | 2 | 85.609 | .975 | .380 |
| Intercept | 42443.351 | 1 | 42443.351 | 483.429 | <.001 |
| Education  practical - theoretical | 58.391 | 1 | 58.391 | .665 | .416 |
| DHH - DLD | 148.866 | 1 | 148.866 | 1.696 | .195 |
| Error | 10886.766 | 124 | 87.797 |  |  |
| Total | 343019.000 | 127 |  |  |  |
| Corrected total | 11057.984 | 126 |  |  |  |

Note: a. R Squared = .015 (Adjusted R Squared = .000). *N* = 127. DHH *n* = 32. DLD *n* = 95.

*4. Statistical Robustness — With unbalanced n and likely heteroscedasticity, use Welch’s t (or preferably linear models with HC3 robust SEs and planned contrasts). Report Hedges’ g with CIs, add robust/nonparametric checks (trimmed-mean t, permutation/bootstrap), and re-estimate mediation/moderation with robust SEs plus covariates.*

We apologize for not including the Welch's *t* and Hedges' *g* correction in the first manuscript. Welch's *t*-test is conducted, and a Hedges' *g* correction was applied, which can be found in the supplemental materials, Tables 16 to 19. The results of both tests did not lead to statistically significant alterations in the findings.

Table 16

*Robust Test of Equality of Means Reference Group - Target Group*

| Welch | Statistic^a^ | df1 | df2 | Sig. |
| --- | --- | --- | --- | --- |
| Child abuse | 15.669 | 1 | 208.091 | <.001 |
| Household dysfunction | 1.454 | 1 | 191.146 | .229 |
| 16 ACEs total | 10.062 | 1 | 202.012 | .002 |
| Stress impact | 25.358 | 1 | 174.093 | <.001 |
| Well-being | 7.341 | 1 | 181.719 | .007 |

Note: a. Asymptotically F distributed. *N* = 213. Adolescents with CP *n* = 127. Reference group, RG *n* = 86. DHH *n* = 32, DLD *n* = 95.

Table 17

*Robust Test of Equality of Means DHH Adolescents - Adolescents with DLD*

| Welch | Statistic^a^ | df1 | df2 | Sig. |
| --- | --- | --- | --- | --- |
| Child abuse | 1.854 | 1 | 59.714 | .178 |
| Household dysfunction | 1.413 | 1 | 68.330 | .239 |
| 16 ACEs total | 1.338 | 1 | 61.365 | .252 |
| Stress impact | 2.557 | 1 | 44.954 | .117 |
| Well-being | 1.601 | 1 | 65.945 | .210 |

Note: a. Asymptotically F distributed. *N* = 127. DHH *n* = 32, DLD *n* = 95.

Table 18

*Independent Samples Effect Sizes Reference Group - Target Group*

|  |  |  | 95% Confidence Interval | |
| --- | --- | --- | --- | --- |
| Hedges'*d* | Standardizer^a^ | Point estimate | Lower | Upper |
| Child abuse | 1.332 | .523 | .245 | .800 |
| Household dysfunction | 1.303 | .165 | -.108 | .438 |
| 16 ACEs total | 3.064 | .426 | .150 | .702 |
| Stress impact | 14.182 | .724 | .425 | 1.021 |
| Well-being | 9.429 | -.378 | -.652 | -.102 |

Note: a. The denominator used in estimating the effect sizes.

Table 19

*Independent Samples Effect Sizes DHH Adolescents - Adolescents with DLD*

|  |  |  | 95% Confidence Interval | |
| --- | --- | --- | --- | --- |
| Hedges'*d* | Standardizer^a^ | Point estimate | Lower | Upper |
| Child abuse | 1.462 | -.260 | -.659 | .140 |
| Household dysfunction | 1.342 | -.213 | -.612 | .186 |
| 16 ACEs total | 3.279 | -.218 | -.617 | .181 |
| Stress impact | 14.802 | -.350 | -.775 | .077 |
| Well-being | 9.414 | .231 | -.169 | .629 |

Note: a. The denominator used in estimating the effect sizes. *N* = 127. DHH *n* = 32, DLD *n* = 95.

In the (moderated) mediation analyses, a heteroscedasticity-consistent standard error and covariate matrix estimator is used (supplementary Tables 14 and 15). These outcomes also did not result in statistically significant changes in the findings.

Table 14

| *Mediation Effect of Stress Impact (PTSD-screener) on Well-Being* | | | | | | |  |
| --- | --- | --- | --- | --- | --- | --- | --- |
| Outcome variable stress impact | | | | | | |  |
| Model summary | | | | | | |  |
|  | *R* | *R*-sq | MSE | *F*(HC4) | df1 | df2 | *p* |
|  | .5074 | .2575 | 167.1385 | 52.5694 | 1.0000 | 188.0000 | .0000 |
|  |  |  |  |  |  |  |  |
| Model |  |  |  |  |  |  |  |
|  | Coeff | se(HC4) | *t* | *p* | LLCI | ULCI |  |
| constant | 14.5498 | 1.8267 | 7.9650 | .0000 | 10.9463 | 18.1532 |  |
| ACEs | 2.5721 | .3547 | 7.2505 | .0000 | 1.8723 | 3.2719 |  |
|  |  |  |  |  |  |  |  |
| Outcome variable Well-being | | | | | | | |
| Model summary | | | | | | | |
|  | *R* | *R*-sq | MSE | *F*(HC4) | df1 | df2 | *p* |
|  | .3063 | .0938 | 82.0666 | 10.3207 | 2.0000 | 187.0000 | .0001 |
|  | | | | | | | |
| Model - Direct effect of ACEs on well-being | | | | | | | |
|  | Coeff | se(HC4) | *t* | *p* | LLCI | ULCI |  |
| constant | 56.9933 | 1.3733 | 41.5000 | .0000 | 54.2841 | 59.7025 |  |
| ACEs | -.1089 | .3076 | -.3540 | .7238 | -.7157 | .4979 |  |
| Stress impact | -.1820 | .0483 | -3.7663 | .0002 | -.2773 | -.0867 |  |
|  |  |  |  |  |  |  |  |
| Direct effect of X (ACEs) on Y (well-being) | | | | | | | |
|  | Effect | se(HC4) | *t* | *p* | LLCI | ULCI |  |
|  | -.1089 | .3076 | -.3540 | .7238 | -.7157 | .4979 |  |
|  |  |  |  |  |  |  |  |
| Indirect effect(s) of X (ACEs) on Y (well-being) | | | | | | |  |
|  | Effect | BootSE | BootLLCI | BootULCI |  |  |  |
| Stress impact | -.4681 | .1412 | -.7730 | -.2169 |  |  |  |
|  |  |  |  |  |  |  |  |
| Bootstrap results for regression model parametrics | | | | | | | |
| Outcome variable stress impact | | | | | | | |
|  | Coeff | BootMean | BootSE | BootLLCI | BootULCI |  |  |
| constant | 14.5498 | 14.4915 | 1.7729 | 11.1049 | 17.9967 |  |  |
| ACEs | 2.5721 | 2.5845 | 3344 | 1.9379 | 3.2396 |  |  |
|  |  |  |  |  |  |  |  |
| Outcome variable well-being | | | | | | | |
|  | Coeff | BootMean | BootSE | BootLLCI | BootULCI |  |  |
| constant | 56.9933 | 57.0045 | 1.3377 | 54.3718 | 59.6678 |  |  |
| ACEs | -.1089 | -.1052 | .2911 | -.6721 | .4599 |  |  |
| Stress impact | -.1820 | -.1828 | .0487 | -.2777 | -.0888 |  |  |

Note: *N* = 190, missing *n* = 23. Adolescents with CP, *n* = 114 (DHH *n* = 28, DLD *n* = 86). Reference group, RG *n* = 76. Level of confidence for all confidence intervals in output: 95.0000. Number of bootstrap samples for percentile bootstrap confidence intervals: 5000. A heteroscedasticity consistent standard error and covariate matrix estimator was used.

Table 15

| *Moderated Mediation - Moderation Effect of Communication Problems on Mediation ACEs, Stress Impact, and Well-being* | | | | | | | |
| --- | --- | --- | --- | --- | --- | --- | --- |
| Outcome variable stress impact | | | | | | |  |
| Model summary | | | | | | |  |
|  | *R* | *R*-sq | MSE | *F*(HC4) | df1 | df2 | *p* |
|  | .5556 | .3087 | 157.2812 | 26.7038 | 3.0000 | 186.0000 | .0000 |
|  |  |  |  |  |  |  |  |
| Model |  |  |  |  |  |  |  |
|  | Coeff | se (HC4) | *t* | *p* | LLCI | ULCI |  |
| constant | 10.9013 | 2.4554 | 4.4398 | .0000 | 6.0573 | 15.7453 |  |
| ACEs | 2.4745 | .6601 | 3.7490 | .0002 | 1.1724 | 3.7767 |  |
| CP-No CP | 8.1175 | 3.6659 | 2.2143 | .0280 | 8853 | 15.3496 |  |
| Int_1 | -.2615 | .8066 | -.3242 | -.7462 | -1.8528 | 1.3298 |  |
|  |  |  |  |  |  |  |  |
| Product terms key Int_1: ACEs x CP-NoCP | | | | | | | |
| Test(s) of highest order unconditional interaction(s) | | | | | | | |
|  | R2-chng | *F*(HC4) | df1 | df2 | *p* |  |  |
| X*W | .0006 | .1051 | 1.0000 | 186.0000 | .7462 |  |  |
|  |  |  |  |  |  |  |  |
| Outcome variable Well-being | | | | | | | |
| Model summary | | | | | | | |
|  | *R* | *R*-sq | MSE | *F*(HC4) | df1 | df2 | *p* |
|  | .3063 | .0938 | 82.0666 | 10.3207 | 2.0000 | 187.0000 | .0001 |
|  | | | | | | | |
| Model | | | | | | | |
|  | Coeff | se (HC4) | *t* | *p* | LLCI | ULCI |  |
| constant | 56.9933 | 1.3733 | 41.5000 | .0000 | 54.2841 | 59.7025 |  |
| ACEs | -.1089 | .3076 | -.3540 | .7238 | -.7157 | .4979 |  |
| Stress impact | -.1820 | .0483 | -3.7663 | .0002 | -.2773 | -.0867 |  |
|  |  |  |  |  |  |  |  |
| Direct effect of X (ACEs) on Y (well-being) | | | | | | | |
|  | Effect | se(HC4) | *t* | *p* | LLCI | ULCI |  |
|  | -.1089 | .3076 | -.3540 | .7238 | -.7157 | .4979 |  |
|  |  |  |  |  |  |  |  |
| Conditional indirect effects of X (ACEs) on Y (well-being) | | | | | | | |
| Indirect effect of ACEs - stress impact - well-being | | | | | | |  |
|  | CP-No CP | Effect | BootSE | BootLLCI | BootULCI |  |  |
|  | .0000 | -.4503 | .1610 | -.8140 | -.1798 |  |  |
|  | 1.0000 | -.4027 | .1285 | -.6841 | -.1824 |  |  |
|  |  |  |  |  |  |  |  |
| Index of moderated mediation (difference between conditional indirect effects): | | | | | | | |
|  |  | Index | BootSE | BootLLCI | BootULCI |  |  |
| CP-No CP |  | .0476 | .1404 | -.2278 | .3413 |  |  |
|  |  |  |  |  |  |  |  |
| Bootstrap results for regression model parameters | | | | | | | |
| Outcome variable Stress impact | | | | | | | |
|  | Coeff | BootMean | BootSE | BootLLCI | BootULCI |  |  |
| Constant | 10.9013 | 10.7840 | 2.3256 | 6.2871 | 15.5671 |  |  |
| ACEs | 2.4745 | 2.5092 | .5952 | 1.3217 | 3.7071 |  |  |
| CP-No CP | 8.1175 | 8.1162 | 3.5231 | .9840 | 14.8691 |  |  |
| Int_1 | -.2615 | -.2711 | .7412 | -1.7059 | 1.2397 |  |  |
|  |  |  |  |  |  |  |  |
| Outcome variable well-being | | | | | | | |
|  | Coeff | BootMean | BootSE | BootLLCI | BootULCI |  |  |
| Constant | 56.9933 | 56.9996 | 1.3718 | 54.3366 | 59.7446 |  |  |
| ACEs | -.1089 | -.1084 | .2920 | -.6882 | .4626 |  |  |
| Stress impact | -.1820 | -.1814 | .0473 | -.2737 | -.0880 |  |  |

Note: *N* = 190, missing *n* = 23. Adolescents with CP, *n* = 114 (DHH *n* = 28, DLD *n* = 86). Reference group, RG *n* = 76. Level of confidence for all confidence intervals in output: 95.0000. Number of bootstrap samples for percentile bootstrap confidence intervals: 5000. A heteroscedasticity consistent standard error and covariate matrix estimator was used.

We hope that our responses and revisions satisfactorily addressed the issues and concerns raised by you, as the editor, and the reviewers. We are pleased to resubmit our revised manuscript for your further consideration.

Sincerely,

Len Martijn, MSc
